# Supplementary material for: Perceived Work Ability during Enforced Working from Home Due to the COVID-19 Pandemic among Finnish Higher Educational Staff
Source: Int J Environ Res Public Health. 2022 May 20;19(10):6230. doi: 10.3390/ijerph19106230 (PMC9140956; doi:10.3390/ijerph19106230)
Supplement: Supplementary file 1 [file ijerph-19-06230-s001.zip › ijerph-1719930-supplementary.pdf]

Supplementary Material

Principal component analysis: functionality of home as work environment & organizational support

A correlational matrix showed high degree of correlation between the items describing home as a functional workspace as well as between the items describing the organizational support. Multiple regression analysis with the least squared method assumes absence of multicollinearity between the independent variables, therefore we used principal component analysis (PCA) to reduce the items into fewer factors. We employed the varimax rotation for the PCA to obtain the best fitting factor solutions, and factor coefficients were used to obtain the factor scores for the selected factors. The number of the selected factors was based on eigenvalues >1 as well as interpretation of scree plot.

Considering Bartlett's test value (605.18; $p < 0.001$) and Kaiser–Meyer–Olkin test value (0.68) obtained for the variables that described functionality of home as a work environment, the data was determined suitable for factor analysis (1). The best fitting solution was a one-factor solution (Supplementary Table 1), which explained 47% of the variation in the included items. Cronbach alpha was 0.69 which shows adequate internal consistency for five included items (2).

Similarly, for the items describing organizational support data was determined suitable for factor analyses as indicated by Bartlett's test value (1185.81; $p < 0.001$) and Kaiser–Meyer–Olkin test value (0.84). Cronbach alpha of 0.82 suggested relatively high internal consistency between the items. Factor analyses of the items describing organizational support supported a one-factor solution that explained 52% of the variation in the included items (Supplementary Table S1).

Supplementary Table S1. Principal component analysis of items describing university employees' adequacy of home as workplace (i) and organizational support (ii) during COVID-19 lockdown

	Factor score coefficients	Factor loadings	Communalities
**Variables describing functionality of home as work environment**			
adequate space at home for remote working	0.34	0.76	0.58
necessary equipment at home for working	0.29	0.64	0.41
enough peace at home for working	0.33	0.74	0.54
ability to maintain a healthy work-life balance	0.31	0.68	0.46
home's internet connection works well enough	0.22	0.50	0.25
**Variance explained, %**	44.7		
**Eigenvalue**	2.23		
**Variables describing organizational support**			
clear communication from the top management about the exceptional circumstances	0.22	0.67	0.45
quick enough responses to practical questions	0.25	0.78	0.60
enough instructions for performing tasks and duties from home	0.25	0.78	0.61
support for work when difficulties encountered	0.25	0.76	0.58
enough instructions for using the electronic systems and tools	0.23	0.72	0.51
the electronic systems and tools have worked well technically	0.19	0.57	0.33
**Variance explained, %**	51.7		
**Eigenvalue**	3.08		

Note. Extraction method principal component analysis. Varimax rotation with Kaiser Normalization. [a]Kaiser-Meyer-Olkin Measure of Sampling Adequacy = 0.68. Bartlett's test p-value <0.001. [b]Kaiser–Meyer–Olkin test value = 0.84. Bartlett's test p-value < 0.001.

Supplementary Table S2. Model fit indices of latent class growth analysis: profiles of work ability during COVID-19 lockdown among university employees (n = 678)

Number of latent classes	AIC	BIC	Class proportions	Entropy	Sample-size adjusted BIC	Estimated posterior probabilities	LMR p-value
2	5959.4	5995.5	0.71/0.29	0.78	5970.1	0.95/0.91	<0.001
3	5748.5	5798.2	0.19/0.16/0.65	0.77	5763.3	0.91/0.92/0.89	<0.001
4	5695.0	5758.3	0.06/0.16/0.55/0.23	0.75	5713.9	0.84/0.88/0.89/0.80	<0.001
5	5662.7	5739.5	0.23/0.02/0.06/0.53/0.16	0.77	5685.5	0.80/0.85/0.87/0.87/0.89	<0.001
**6**	**5650.8**	**5741.2**	**0.13/0.06/0.04/0.53/0.23/0.02**	**0.79**	**5677.7**	**0.85/0.88/0.73/0.88/0.82/0.84**	**0.03**
7	5647.6	5751.5	0.05/0.13/0.02/0.04/0.06/0.51/0.19	0.77	5678.5	0.58/0.86/0.83/0.73/0.87/0.89/0.75	0.159
Quadratic term included							
q4	5697.7	5779.0	0.16/0.06/0.23/0.55	0.76	5721.9	0.86/0.84/0.81/0.89	<0.001
q5	5661.3	5760.7	0.53/0.06/0.02/0.16/0.23	0.78	5690.8	0.87/0.86/0.86/0.89/0.81	<0.001
q6	5652.7	5770.2	0.51/0.02/0.06/0.11/0.23/0.06	0.78	5687.6	0.86/0.84/0.83/0.84/0.82/0.87	0.168

AIC= Akaike Information Criterion, BIC= Bayesian Information Criterion, LMR= Lo-Mendell Rubin

References:

1. Sharma (1996) Sharma S. Applied multivariate techniques. John Wiley & Sons, Inc; New York: 1996. p. 493.

2. Pallant, J. (2013). SPSS survival manual. McGraw-Hill Education (UK). p. 97-101.
